# Tumor deposit serves as a prognostic marker in gastric cancer: A propensity score‐matched analysis comparing survival outcomes

**DOI:** 10.1002/cam4.2963

**Published:** 2020-03-12

**Authors:** Liang Wenquan, Liu Yuhua, Cui Jianxin, Xi Hongqing, Zhang Kecheng, Li Jiyang, Gao Yunhe, Liu Yi, Zhang Wang, Li Shaoqing, Lu Yixun, Qiao Shen, Xue Wanguo, Qiao Zhi, Chen Lin

**Affiliations:** ^1^ Department of General Surgery & Institute of General Surgery Chinese PLA General Hospital Beijing China; ^2^ Medical School of Chinese PLA Beijing China; ^3^ Institute of Army Hospital Management Chinese People’s Liberation Army General Hospital Beijing China; ^4^ Medical Big Data Center Chinese People’s Liberation Army General Hospital Beijing China

**Keywords:** gastric cancer, staging, survival, tumor deposit

## Abstract

**Background:**

Gastric cancer (GC) treatment is determined by accurate tumor staging. The value of tumor deposit (TD) in prognostic prediction staging system is not yet determined.

**Methods:**

We retrospectively analyzed clinical information on GC patients who underwent gastrectomy at the Department of General Surgery of the Chinese PLA General Hospital from July 2014 to June 2016. Propensity score matching (PSM) was performed to reduce the possibility of selection bias according to the presence of TD.

**Results:**

Of the 1034 GC patients, 240 (23.21%) presented with TD, which was associated with younger age and larger tumor size (all *P* < .05). TD‐positive patients had a worse survival than TD‐negative patients before (*P* < .001) and after (*P* = .017) matching. Multivariable analysis showed that mortality risk of patients with TD increased by 58%, 62%, 37%, and 40% in the crude (HR = 1.58, 95% CI 1.32‐1.89, *P* < .001), adjusted I (HR = 1.62, 95% CI 1.35‐1.94, *P* < .001), adjusted II (HR = 1.37, 95% CI 1.13‐1.66, *P* = .001), and adjusted III (HR = 1.40, 95% CI 1.16‐1.68, *P* < .001) models before matching. Similarly, in the PSM cohort patients with TD had worse prognosis in the crude (HR = 1.32, 95% CI 1.07‐1.63, *P* = .011), adjusted I (HR = 1.35, 95% CI 1.09‐1.67, *P* = .005), adjusted II (HR = 1.26, 95% CI 1.00‐1.58, *P* = .049), and adjusted III (HR = 1.33, 95% CI 1.07‐1.65, *P* = .010) models. TD had a similar value range between N1 and N2 stages among different models.

**Conclusions:**

Among GC patients, TD is associated with survival and may have a role in the staging of patients.

## Graphical abstract

Kaplan‐Meier curves for TD negative and positive gastric patients before and after propensity score matching.

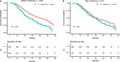

## 1 INTRODUCTION

Gastric cancer (GC) is one of the deadliest upper digestive tumors and is the second leading cause of cancer‐related death in the world.^1^ The accuracy of cancer staging is considered to be a cornerstone in the treatment of cancers. The American Joint Committee on Cancer (AJCC) tumor‐node‐metastasis (TNM) classification is applied internationally for many tumor staging including GC.^2^, ^3^ With advancements in diagnostic medicine and increasing treatment possibilities, the TNM staging system for GC is updated regularly. However, to guide new treatment choices and enable a better prediction of survival for GC, more detailed staging strategies are required.

Tumor deposit (TD) was first described as mesenteric satellites of colorectal cancer in 1935.^4^, ^5^ It was commonly defined as discontinuous macroscopic or microscopic deposits from the primary tumor and without any residual lymph node structures.^6^ Beginning with the fifth edition, TD was incorporated in the TNM staging manuals of colorectal cancer and evolved to the eighth edition as N1c categories.^7^ In GC, TD is also frequently observed, although few studies have investigated its prognostic effects. Thus, it is necessary to demonstrate whether there is a place for TD in the staging of patients with GC and also address the many questions regarding the definition and reproducibility of this category in staging.

Propensity score matching (PSM) analysis becomes more widely accepted and used, especially in some studies where random assignment is not appropriate.^8^ In the last few years, PSM has increasingly been used to reduce the bias between matched arms in observational studies.^9^, ^10^, ^11^, ^12^, ^13^

This study aimed to investigate the prognostic effect of TD in GC patients. Moreover, this study compared the regional lymph node stage and TD to proposed appropriate revisions for accurate tumor staging. In addition, to improve the robustness of this study, PSM techniques were adopted.

## 2 PATIENTS AND METHODS

### 2.1 Study population

**Figure 1:**
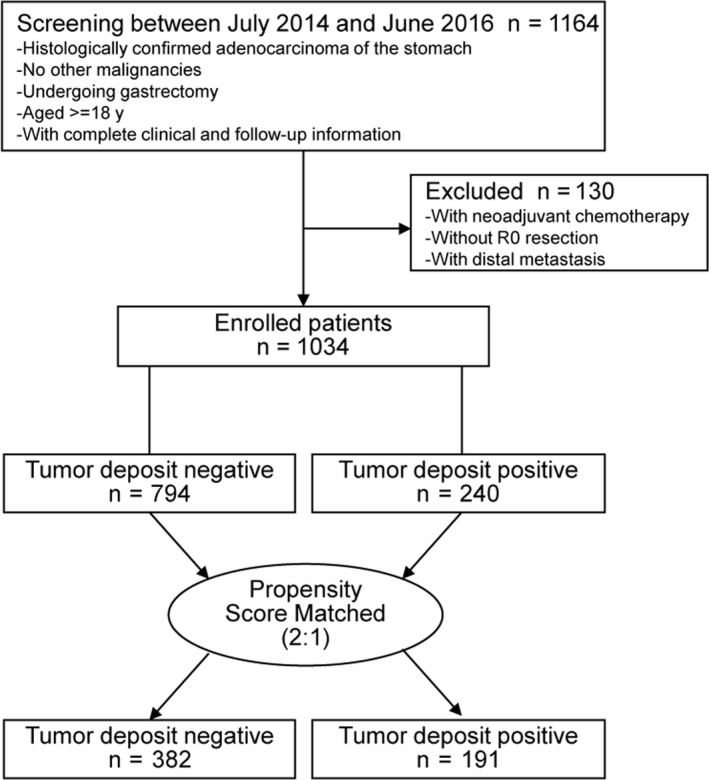
Flow chart

Clinical information on all patients with GC who underwent gastrectomy at the Department of General Surgery of the Chinese PLA General Hospital from July 2014 to June 2016 was retrospectively collected and analyzed. The inclusion criteria were patients (a) with histologically confirmed adenocarcinoma of the stomach, (b) with no other malignancies, (c) undergoing gastrectomy, (d) aged ≥ 18 years, and (e) with complete clinical information and follow‐up. The exclusion criteria included patients (a) with neoadjuvant chemotherapy, (b) without R0 resection, and (c) with distal metastasis. Of the 1164 participants who were screened, 130 were excluded resulting in 1034 patients being included in the study. The flow chart of the patient selection process is presented in Figure 1. Participants’ informed consent was not required for this study because of its retrospective nature. The Institutional Review Boards of the Chinese PLA General Hospital approved this study and the study was conducted in accordance with the Declaration of Helsinki.

### 2.2 Definition of TD

**Figure 2:**
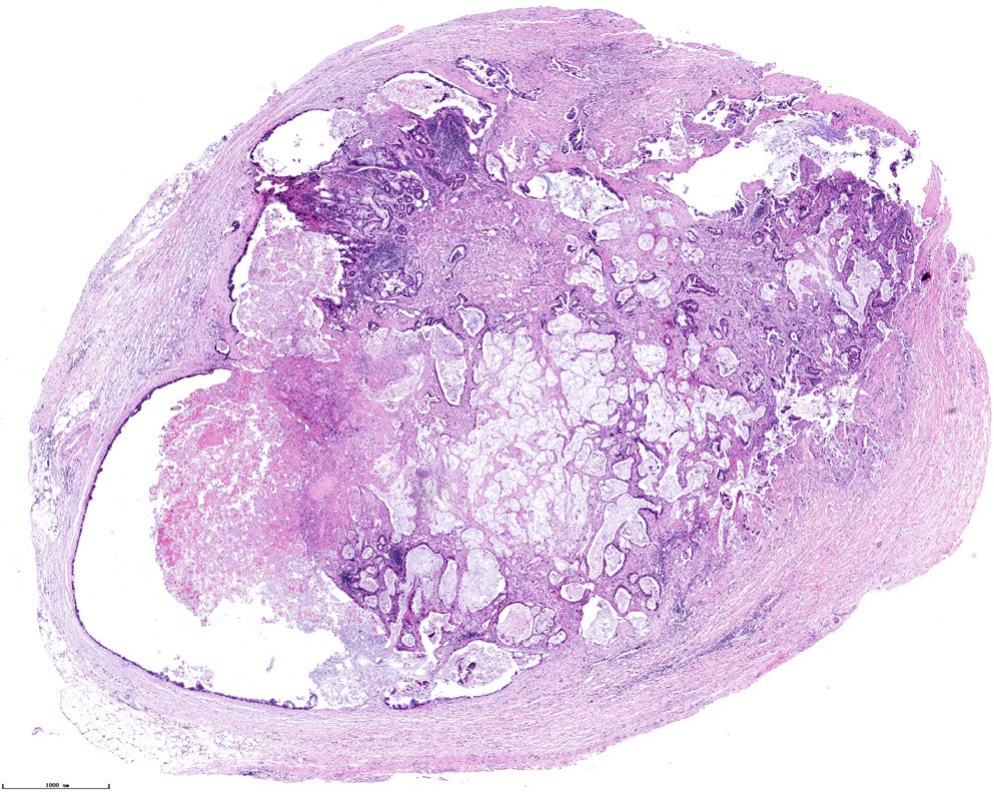
An example of a tumor deposit (TD) of gastric cancer to show the pathological features

The histological sections of tumor specimens were reviewed independently by two pathologists, and disagreements were confirmed by a third expert. The definition of TD was according to the chapter of stomach tumor in the AJCC Cancer Staging Manual (Eighth Edition).^14^ Positive TD is defined as discrete tumor nodules within the lymph drainage area of the primary carcinoma without identifiable lymph node tissue or identifiable vascular or neural structure. An example of the pathological illustration for TD is shown in Figure 2.

### 2.3 Evaluation of clinicopathologic variables

The following features of enrolled patients were obtained: age, sex, tumor size, tumor location, surgical method, gastrectomy, lymphovascular invasion, perineural invasion, histologic grade, depth of invasion, lymph node metastases (LNM), and the TD status. Age and sex of the patients were collected from inpatient medical records. Data on tumor size, tumor location, surgical method, and gastrectomy were obtained from operative reports. Tumor sizes were defined as the maximum diameter. Data on lymphovascular invasion, perineural invasion, histologic grade, depth of invasion, and LNM were recorded from the pathology reports and classified based on the Japanese classification of gastric carcinoma.^15^ Histologic grade subtypes were classified into two groups: differentiated type and undifferentiated type.^16^, ^17^

### 2.4 Follow‐up

The primary endpoint of the cohort was overall survival (OS) and follow‐up of the entire population was measured from the date of surgery to the time of last follow‐up (June 2019) or date of all‐cause death. We performed the follow‐up every 3 months for the first year after surgery and thereafter every 6 months. The median follow‐up was 29 (range 4‐59) months and follow‐up data were managed by the second author and stored in the hospital electronic medical record system.

### 2.5 Statistical analysis

Categorical variables are showed as frequency and continuous variables are presented as mean and standard deviation (SD). The ratio of TD negative and positive is 3.3 (794/240) before matching. The factors^8^, ^18^ such as (1) sufficient sample size as possible after matching, (2) reducing the inherent differences between the selected TD‐negative and TD‐positive groups, and (3) maximum statistical power, a 2:1 matching ratio were used in the present study. Variables including age, sex, tumor size, tumor location, surgical method, gastrectomy, lymphovascular invasion, perineural invasion, histologic grade, depth of invasion, and LNM were used as the matching criteria and the caliper was set at 0.01. Patients’ characteristics were evaluated before and after matching. Univariable survival analysis was conducted by Kaplan‐Meier and Log‐Rank test to detect the relation between variables and OS. The status of LNM and TD was included in the multivariate analysis by Cox Proportional Hazards Regression. Four models were constructed in the multivariate analysis to identify the independent effect of TD on the prognosis of GC: crude model, no covariates were adjusted; adjusted I model, only sociodemographic data (age and sex) were adjusted; adjusted model II, covariates were elected for the fully adjusted if the matched odds ratio changed at least 10% as the result of adding those covariates, which was described in previous studies^19^; adjusted model II, covariates were adjusted if they were significant in the univariate analysis. The statistical software packages R and Empower Stats (Boston, MA, USA) were used for the Statistical Analysis. *P* < .05 were considered statistically significant.

## 3 RESULTS

### 3.1 Participant characteristics

The demographic and pathologic characteristics of the cohort were summarized according to TD status. As shown in Table 1, of the 1034 GC patients, 794 (76.79%) patients were TD negative, whereas 240 (23.21%; ratio, 3.3:1) were TD positive. TD‐positive patients were significantly younger and had a larger tumor size (all *P* < .05) than TD‐negative patients. More patients received laparoscopic surgery in the TD‐positive group (78.33%) than in the TD‐negative group (57.05%). The incidence of lymphovascular invasion was higher among TD‐positive patients (38.75%) than among negative patients (27.33%). More TD‐positive patients harbored advanced pathological T4a (62.50% vs 55.54%), T4b (20.83% vs 10.96%), and N3a (34.17% vs 20.40%) stages than TD‐negative patients. No significant differences were detected between the presence and absence of TD in other clinicopathologic characteristics including sex, tumor location, gastrectomy, and perineural invasion. After 2:1 PSM, 382 TD‐negative and 191 TD‐positive GC patients were screened out for sensitivity analysis. After matching, the two groups divided by TD status were well balanced in all of the variables (all *P* < .05).

**Table 1:** Baseline characteristics of TD‐negative and TD‐positive gastric cancer patients before and after propensity score matching

Characteristics	Before matching n = 1034			After matching n = 573		
	TDs (−) n = 794	TDs (+) n = 240	*P* value	TDs (−) n = 382	TDs (+) n = 191	*P* value
Age, years, mean (SD)	62.26 ± 11.00	59.96 ± 11.69	.005	61.92 ± 10.58	61.51 ± 11.41	.672
Gender			.374			.220
Male	611 (76.95%)	178 (74.17%)		280 (73.30%)	149 (78.01%)	
Female	183 (23.05%)	62 (25.83%)		102 (26.70%)	42 (21.99%)	
Tumor size, cm, mean (SD)	5.83 ± 3.60	6.22 ± 2.72	<.001	6.26 ± 4.05	6.01 ± 2.46	.436
Tumor location			.324			.314
Upper	228 (28.72%)	69 (28.75%)		101 (26.44%)	64 (33.51%)	
Middle	138 (17.38%)	46 (19.17%)		72 (18.85%)	30 (15.71%)	
Lower	343 (43.20%)	91 (37.92%)		165 (43.19%)	74 (38.74%)	
Two regions or entire	85 (10.71%)	34 (14.17%)		44 (11.52%)	23 (12.04%)	
Surgical method			<.001			.737
Open surgery	341 (42.95%)	52 (21.67%)		99 (25.92%)	52 (27.23%)	
Laparoscopic surgery	453 (57.05%)	188 (78.33%)		283 (74.08%)	139 (72.77%)	
Gastrectomy			.134			.485
Proximal	223 (28.09%)	58 (24.17%)		93 (24.35%)	55 (28.80%)	
Distal	327 (41.18%)	92 (38.33%)		156 (40.84%)	76 (39.79%)	
Total	244 (30.73%)	90 (37.50%)		133 (34.82%)	60 (31.41%)	
Lymphovascular invasion			<.001			.898
Negative	577 (72.67%)	147 (61.25%)		264 (69.11%)	133 (69.63%)	
Positive	217 (27.33%)	93 (38.75%)		118 (30.89%)	58 (30.37%)	
Perineural invasion			.139			.579
Negative	597 (75.19%)	169 (70.42%)		294 (76.96%)	143 (74.87%)	
Positive	197 (24.81%)	71 (29.58%)		88 (23.04%)	48 (25.13%)	
Histologic grade			.849			.809
Differentiated	333 (41.94%)	99 (41.25%)		154 (40.31%)	75 (39.27%)	
Undifferentiated	461 (58.06%)	141 (58.75%)		228 (59.69%)	116 (60.73%)	
Depth of invasion			<.001			.974
T1	58 (7.30%)	6 (2.50%)		11 (2.88%)	6 (3.14%)	
T2	101 (12.72%)	13 (5.42%)		25 (6.54%)	13 (6.81%)	
T3	107 (13.48%)	21 (8.75%)		43 (11.26%)	18 (9.42%)	
T4a	441 (55.54%)	150 (62.50%)		254 (66.49%)	130 (68.06%)	
T4b	87 (10.96%)	50 (20.83%)		49 (12.83%)	24 (12.57%)	
Lymph node metastasis			<.001			.062
N0	212 (26.70%)	31 (12.92%)		80 (20.94%)	30 (15.71%)	
N1	153 (19.27%)	34 (14.17%)		64 (16.75%)	32 (16.75%)	
N2	159 (20.03%)	67 (27.92%)		92 (24.08%)	57 (29.84%)	
N3a	162 (20.40%)	82 (34.17%)		95 (24.87%)	58 (30.37%)	
N3b	108 (13.60%)	26 (10.83%)		51 (13.35%)	14 (7.33%)	

Note

Data are presented as mean ± SD or n (%).

Abbreviations: SD, standard deviation; TD, tumor deposit.

### 3.2 Association between clinicopathologic features and OS

**Figure 3:**
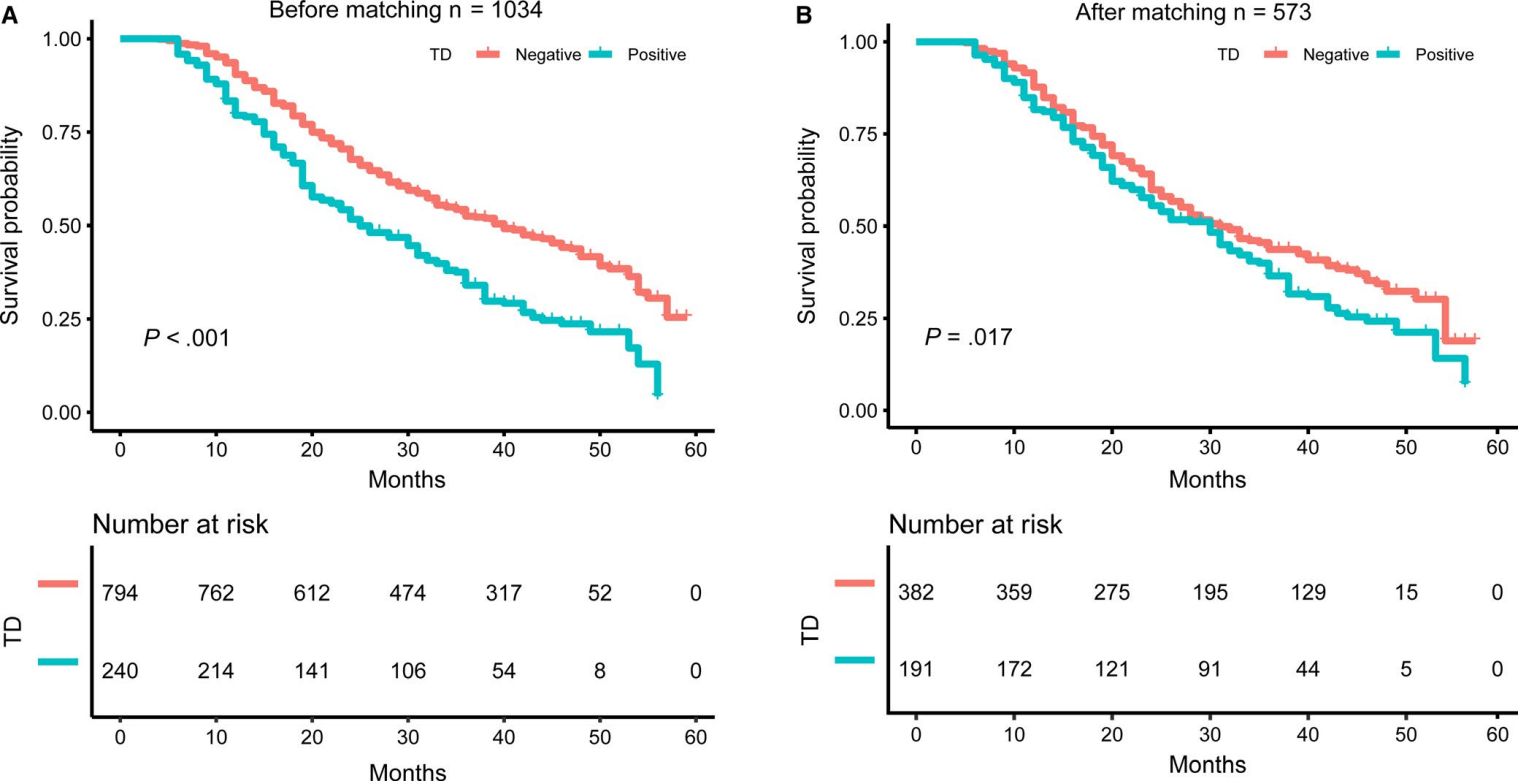
Kaplan‐Meier curves for TD‐negative and TD‐positive gastric patients before and after propensity score matching. (A) Kaplan‐Meier curves for TD‐negative and TD‐positive gastric patients before propensity score matching. (B) Kaplan‐Meier curves for TD‐negative and TD‐positive gastric patients after propensity score matching. Abbreviation: TD tumor deposit

The univariate analysis of the prognostic factors before and after PSM is shown in Table 2. Older age was significantly associated with reduced OS after PSM (hazard ratio [HR] = 1.01, 95% confidence interval [CI] 1.00‐1.02, *P* = .013) but null associations were observed before PSM (HR = 1.01, 95% CI 1.00‐1.01, *P* = .121). Larger tumor size (before PSM, HR = 1.10, 95% CI 1.08‐1.12, *P* = .013) and two regions or entire stomach infiltration significantly increased the risk of mortality both in the whole cohort and in the PSM cohort (all *P* < .05). Laparoscopic surgery was associated with increased mortality risk before PSM (HR = 1.23, 95% CI 1.04‐1.45, *P* = .014) but protective (HR = 0.79, 95% CI 0.63‐0.99, *P* = .037) after PSM, which requires further analysis. Patients who underwent total gastrectomy had a higher mortality risk both in the whole cohort (HR = 1.75, 95% CI 1.42‐2.15, *P* < .001) and in the PSM cohort (HR = 1.51, 95% CI 1.16‐1.96, *P* = .002) than those who underwent proximal gastrectomy. In terms of pathologic features, lymphovascular invasion, perineural invasion, and undifferentiated histologic grade were associated with reduced OS both in the whole cohort and in the PSM cohort (all *P* < .05). The survival time of GC patients decreased with the increase in the T stage and N stage in the whole cohort and the PSM cohort. Patients in the T2, T3, T4a, T4b stages had 1.99 (95% CI 0.68‐5.87; *P* = .209), 2.54 (95% CI 0.91‐7.13; *P* = .076), 3.66 (95% CI 1.36‐9.82; *P* = .010), 5.53 (95% CI 2.01‐15.22; *P* = .001) times increased mortality risk compared to those in the T1 stage, respectively, after matching. In addition, the risk of mortality in GC patients in the N1, N2, N3a, N3b stages was 1.06 (95% CI 0.71‐1.59; *P* = .772), 1.86 (95% CI 1.33‐2.61; *P* < .001), 3.46 (95% CI 2.49‐4.80; *P* < .001), and 7.81 (95% CI 5.33‐11.46; *P* < .001) times higher than those without LNM after matching. Similar results for the T stage and N stage were observed before matching. There was a significant difference between the mortality risk for patients with and without TD in the whole cohort (HR 1.75, 95% CI 1.42‐2.08; *P* < .001) and in the PSM cohort (HR 1.28, 95% CI 1.04‐1.58; *P* = .019). Kaplan‐Meier survival curves for the patients stratified by their TD status are presented in Figure 3. Significant differences in survival were observed between TD‐positive and TD‐negative categories before (Figure 3A, *P* < .001) and after (Figure 3B, *P* = .017) matching.

**Table 2:** Univariable cox proportional hazards analysis for overall survival of gastric cancer patients before and after propensity score matching

Characteristics	Before matching n = 1034		After matching n = 573	
	Statistics	HR (95% CI) *P* value	Statistics	HR (95% CI) *P* value
Age, years, mean (SD)	61.72 ± 11.20	1.01 (1.00, 1.01) .121	61.78 ± 10.86	1.01 (1.00, 1.02) .013
Gender				
Male	789 (76.31%)	1.00 (Ref.)	429 (74.87%)	1.00 (Ref.)
Female	245 (23.69%)	1.11 (0.92, 1.33) .279	144 (25.13%)	1.10 (0.88, 1.38) .401
Tumor size, cm, mean (SD)	5.92 ± 3.42	1.10 (1.08, 1.12) <.001	6.18 ± 3.60	1.08 (1.06, 1.11) <.001
Tumor location				
Upper	297 (28.72%)	1.00 (Ref.)	165 (28.80%)	1.00 (Ref.)
Middle	184 (17.79%)	1.25 (0.99, 1.58) .066	102 (17.80%)	1.03 (0.76, 1.39) .854
Lower	434 (41.97%)	0.98 (0.80, 1.20) .845	239 (41.71%)	0.89 (0.69, 1.14) .358
Two regions or Entire	119 (11.51%)	2.64 (2.06, 3.38) <.001	67 (11.69%)	2.00 (1.45, 2.75) <.001
Surgical method				
Open surgery	393 (38.01%)	1.00 (Ref.)	151 (26.35%)	1.00 (Ref.)
Laparoscopic surgery	641 (61.99%)	1.23 (1.04, 1.45) .014	422 (73.65%)	0.79 (0.63, 0.99) .037
Gastrectomy				
Proximal	281 (27.18%)	1.00 (Ref.)	148 (25.83%)	1.00 (Ref.)
Distal	419 (40.52%)	1.15 (0.93, 1.41) .193	232 (40.49%)	1.14 (0.88, 1.49) .324
Total	334 (32.30%)	1.75 (1.42, 2.15) <.001	193 (33.68%)	1.51 (1.16, 1.96) .002
Lymphovascular invasion				
Negative	724 (70.02%)	1.00 (Ref.)	397 (69.28%)	1.00 (Ref.)
Positive	310 (29.98%)	2.06 (1.75, 2.42) <.001	176 (30.72%)	2.01 (1.63, 2.48) <.001
Perineural invasion				
Negative	766 (74.08%)	1.00 (Ref.)	437 (76.27%)	1.00 (Ref.)
Positive	268 (25.92%)	1.41 (1.19, 1.67) <.001	136 (23.73%)	1.45 (1.16, 1.82) .001
Histologic grade				
Differentiated	432 (41.78%)	1.00 (Ref.)	229 (39.97%)	1.00 (Ref.)
Undifferentiated	602 (58.22%)	1.56 (1.33, 1.84) <.001	344 (60.03%)	1.53 (1.24, 1.89) <.001
Depth of invasion				
T1	64 (6.19%)	1.00 (Ref.)	17 (2.97%)	1.00 (Ref.)
T2	114 (11.03%)	1.86 (1.01, 3.43) .047	38 (6.63%)	1.99 (0.68, 5.87) .209
T3	128 (12.38%)	3.32 (1.86, 5.92) <.001	61 (10.65%)	2.54 (0.91, 7.13) .076
T4a	591 (57.16%)	4.93 (2.88, 8.43) <.001	384 (67.02%)	3.66 (1.36, 9.82) .010
T4b	137 (13.25%)	9.13 (5.23, 15.96) <.001	73 (12.74%)	5.53 (2.01, 15.22) .001
Lymph node metastasis				
N0	243 (23.50%)	1.00 (Ref.)	110 (19.20%)	1.00 (Ref.)
N1	187 (18.09%)	1.35 (0.99, 1.85) .056	96 (16.75%)	1.06 (0.71, 1.59) .772
N2	226 (21.86%)	2.44 (1.86, 3.20) <.001	149 (26.00%)	1.86 (1.33, 2.61) <.001
N3a	244 (23.60%)	4.69 (3.62, 6.08) <.001	153 (26.70%)	3.46 (2.49, 4.80) <.001
N3b	134 (12.96%)	9.24 (6.96, 12.27) <.001	65 (11.34%)	7.81 (5.33, 11.46) <.001
TD				
Negative	794 (76.79%)	1.00 (Ref.)	382 (66.67%)	1.00 (Ref.)
Positive	240 (23.21%)	1.75 (1.47, 2.08) <.001	191 (33.33%)	1.28 (1.04, 1.58) .019

Note

Data are presented as mean ± SD or n (%).

Abbreviations: Ref, reference; SD, standard deviation; TD, tumor deposit.

### 3.3 Multivariable analysis of LNM and TD

Placement of TD in the nodal category for GC patients and the category in which TD should be equal are two important issues for clinical research. To further compare the prognostic impact of LNM and TD, a Cox multivariate analysis of these two variables was performed (Table 3). We constructed four models to detect the independent effects of TD on survival including crude model, minimally adjusted (adjusted I) model, fully adjusted (adjusted II) model, and univariate‐related‐factor adjusted (adjusted III) model. The results of the multivariate analyses showed that the survival time of GC patients decreased with the increase in the N stage before and after matching. Regardless of the N stage, mortality risk of patients with TD increased by 58%, 62%, 37%, and 40% in the crude model (HR = 1.58, 95% CI 1.32‐1.89, *P* < .001), adjusted I model (HR = 1.62, 95% CI 1.35‐1.94, *P* < .001), adjusted II model (HR = 1.37, 95% CI 1.13‐1.66, *P* = .001), and adjusted III model (HR = 1.40, 95% CI 1.16‐1.68, *P* < .001), respectively, before matching. Additionally, similar results were found in the PSM cohort where patients with TD had a worse prognosis in the crude (HR = 1.32, 95% CI 1.07‐1.63, *P* = .011), adjusted I model (HR = 1.35, 95% CI 1.09‐1.67, *P* = .005) adjusted II model (HR = 1.26, 95% CI 1.00‐1.58, *P* = .049), and adjusted III model (HR = 1.33, 95% CI 1.07‐1.65, *P* = .010), respectively. We further compared the prognostic value between every N category and TD and found that TD had a similar value range between N1 and N2 stages among the different models.

**Table 3:** Multivariable cox proportional hazards analysis for overall survival of gastric cancer patients in adjusted models before and after propensity score matching

Characteristics	Crude model	Adjusted I model	Adjusted II model	Adjusted III model
Before matching n = 1034, HR (95% CI) *P* value				
Lymph node metastasis				
N0	1.00 (Ref.)	1.00 (Ref.)	1.00 (Ref.)	1.00 (Ref.)
N1	1.30 (0.95, 1.78) .095	1.27 (0.93, 1.73) .139	0.99 (0.72, 1.36) .941	1.00 (0.72, 1.38) .996
N2	2.27 (1.73, 2.99) <.001	2.23 (1.69, 2.93) <.001	1.76 (1.33, 2.34) <.001	1.76 (1.32, 2.34) <.001
N3a	4.24 (3.26, 5.52) <.001	4.29 (3.29, 5.58) <.001	3.42 (2.60, 4.50) <.001	3.37 (2.56, 4.44) <.001
N3b	9.27 (6.98, 12.32) <.001	9.22 (6.94, 12.26) <.001	5.33 (3.76, 7.56) <.001	5.15 (3.75, 7.09) <.001
TD				
Negative	1.00 (Ref.)	1.00 (Ref.)	1.00 (Ref.)	1.00 (Ref.)
Positive	1.58 (1.32, 1.89) <.001	1.62 (1.35, 1.94) <.001	1.37 (1.13, 1.66) .001	1.40 (1.16, 1.68) <.001
After matching n = 573, HR (95% CI) P value				
Lymph node metastasis				
N0	1.00 (Ref.)	1.00 (Ref.)	1.00 (Ref.)	1.00 (Ref.)
N1	1.04 (0.70, 1.56) .841	1.00 (0.67, 1.51) .982	0.96 (0.64, 1.45) .845	1.01 (0.66, 1.53) .967
N2	1.82 (1.30, 2.55) <.001	1.79 (1.27, 2.51) <.001	1.79 (1.27, 2.52) .001	1.78 (1.26, 2.52) .001
N3a	3.35 (2.41, 4.66) <.001	3.35 (2.41, 4.66) <.001	3.30 (2.35, 4.63) <.001	3.45 (2.44, 4.87) <.001
N3b	8.01 (5.45, 11.76) <.001	7.53 (5.11, 11.08) <.001	4.77 (3.05, 7.47) <.001	5.39 (3.51, 8.27) <.001
TD				
Negative	1.00 (Ref.)	1.00 (Ref.)	1.00 (Ref.)	1.00 (Ref.)
Positive	1.32 (1.07, 1.63) .011	1.35 (1.09, 1.67) .005	1.26 (1.00, 1.58) .049	1.33 (1.07, 1.65) .010

Note

Data are presented as HR (95% CI) P value. Crude model did not adjust covariant; Adjusted I model minimally adjusted for age and gender; Adjusted II model fully adjusted for age, gender, tumor size, tumor location, lymphovascular invasion, and depth of invasion; Adjusted III model fully adjusted for tumor size, tumor location, surgical method, gastrectomy, lymphovascular invasion, perineural invasion, histologic grade, and depth of invasion.

Abbreviations: CI, confidence interval; HR, hazard ratio; TD, tumor deposit; Ref, reference.

## 4 DISCUSSION

We identified that the incidence of TD was 23.21% in a sample of 1034 GC patients in the present study. The presence of TD was associated with younger age, larger tumor size, lymphovascular invasion, and advanced T and N stages. PSM was performed in this study and 382 TD‐negative and 191 TD‐positive GC patients were screened out for sensitivity analysis. Larger tumor size, two regions or entire stomach infiltration, total gastrectomy, lymphovascular invasion, perineural invasion, undifferentiated histologic grade, advanced T and N stages, and presence of TD were significantly associated with increased risk of mortality before and after PSM. Kaplan‐Meier survival curves showed significant survival differences according to the status of TD in both the whole and PSM cohorts. The multivariable analysis further confirmed that TD was a significant predictor of mortality. After adjustment for confounders in different models, the association remained the same before and after PSM. We found that TD had a similar predictive value between N1 and N2 stages and the associations persisted after adjustment of potential confounders. These findings, if further confirmed in multi‐center studies would help identify the value of TD and increase the accuracy of the staging system of GC.

A recent systematic review that included data from 7445 GC patients showed that the incidence of TD ranged from 10.6% to 36.7% (mean 20.9%) and TD was an independent predictor of prognosis in patients with GC,^20^ which is similar to our findings. The relation between TD and other known poor prognostic characteristics might partly explain this observation. For example, TD has been more frequently observed in cancers of large tumor size, poorly differentiated histology, lymphovascular and perineural invasion, advanced T and N stages.^21^, ^22^, ^23^ In this study, TD was associated with poor survival of GC patients by univariate analysis in the whole cohort and PSM cohort which is consistent with previous studies.^21^, ^22^, ^23^, ^24^, ^25^

Many studies have reported that TD is an important prognostic factor for GC.^16^, ^20^, ^21^, ^22^, ^23^, ^24^, ^25^, ^26^ However, this is not yet considered in the current TNM classification of GC. For colorectal cancer, the importance of TD has been acknowledged and been incorporated in the N category in recent editions of TNM classification since 1997.^27^ One of the challenges is whether TD should be added as an independent prognostic factor and where should it be placed in the current TNM categories. There are several hypotheses regarding the inclusion of TD in GC staging in recent years. The theory of incorporating TD into the N category is still the most popular hypothesis. Japanese Gastric Cancer Association also suggested TD be counted as LNM as an experienced‐based suggestion without high‐quality clinical evidence.^15^ A recent study from two Chinese centers regarded TD as LNM in the eighth TNM staging system and the modified N classification was found to be more accurate for the prognostic prediction.^16^ The same theory also is investigated by incorporating TD into the revised N stage in another Chinese single‐center study.^23^ However, arguments were also made from several studies that TD could not be equated to LNM from a biological perspective.^28^, ^29^ One previous study classified TD into five patterns according to different histological features: separate nodular, perivascular, perineural, lymphatic, and endovascular patterns,^30^ which meant differential in origin resulting in different prognostic effects. Another hypothesis recommends TD as the T4 category in view of its origin from perigastric regions.^22^, ^26^, ^30^ In those studies, TD was considered as a form of serosal invasion and peritoneal seeding from the primary tumor. In a study by Anup et al that included 1250 GC patients,^22^ the stage T4 survival rate was very similar to those patients with positive TD. In a study by Sun et al, the TD‐positive GC patients in T1‐4a category had similar prognosis compared to TD‐negative patients in the T4a category.^26^ Recently, studies also put forward the hypothesis that TD positive should be distant metastasis.^30^, ^31^ In these studies, TD was an independent predictor of distant metastasis^30^ and associated with liver and peritoneal metastasis.^31^

TNM classification of colorectal cancer has included TD in the N category as N1c since the seventh edition.^6^ In GC staging, many studies also suggest incorporating TD into the N staging category.^16^, ^21^, ^23^ It is necessary to compare prognostic value between the TD and N category to further understand the staging effects of TD in GC. In the present study, four models were constructed to detect this prognostic effect in multivariable analyses. After adjusting the staging effects of N stage, results of multivariable analyses showed that TD was an independent prognostic factor in GC and had staging effects similar to someplace between N1 and N2 stages. These results were stable in the whole PSM cohorts. Indeed, a complete dissection of regional lymphatic areas may be the precondition to analysis of the staging value of TD and the origin, size, number, and location of TD should be comprehensively considered.^32^

The present study did not aim to provide a novel staging strategy including TD for GC but to examine the prognostic effect of TD. The classical assessment of TNM staging categories was not sufficient and the staging principles of GC should be reconsidered. Our study has some strengths. PSM was implemented to reduce the possibility of selection bias using a logistic regression model. Despite the use of PSM, the non‐interventional nature of our study means that we cannot rule out residual confounding. There are several limitations that require further discussion. First, the findings of this retrospective study from a single Chinese institution may not be generalizable to other settings. Second, only the presence and absence of TD were recorded in this study, whereas other pathologic characteristics such as size and location were not regularly recorded in the histopathology reports. Therefore, these findings should be considered only for hypothesis generation and require additional validation with more extensive studies.

In conclusion, the present study demonstrates that TD is frequently observed and is an indicator of the aggressive characteristics of GC. The presence of TD is a strong and independent prognostic factor and has staging effects similar to someplace between N1 and N2 stages, indicating that TD should be incorporated into staging strategies in GC.

## CONFLICT OF INTEREST

The authors declare that they have no conflict of interest.

## AUTHOR CONTRIBUTIONS

Liang Wenquan, Liu Yuhua, and Cui Jianxin: Paper writing and data analysis; Xi Hongqing, Zhang Kecheng, and Li Jiyang: Clinical and pathological data collection; Gao Yunhe, Liu Yi, Zhang Wang, Li Shaoqing, and Lu Yixun: Patient follow‐up; Qiao Shen and Xue Wanguo: Database establishment; Qiao Zhi and Chen Lin: Supervision and paper revision.

## ETHICAL APPROVAL STATEMENT

The Institutional Review Boards of the Chinese PLA General Hospital approved this study and the study was conducted in accordance with the Declaration of Helsinki.

## Data Availability

The data that support the findings of this study are available from the corresponding author upon reasonable request.
